# 3,3,12,12-Tetra­methyl-1,5,10,14-tetra­oxa­dispiro­[5.2.5.2]hexa­deca­ne

**DOI:** 10.1107/S1600536809021291

**Published:** 2009-06-13

**Authors:** Yong-Jun He, Min-Hao Xie, Pei Zou, Ya-Ling Liu, Jun Wu

**Affiliations:** a Jiangsu Institute of Nuclear Medicine, Wuxi 214063, People’s Republic of China

## Abstract

The mol­ecule of the title compound, C_16_H_28_O_4_, is centrosymmetric. The cyclo­hexane ring and both six-membered dioxane rings adopt chair conformations. In the crystal, the mol­ecules lie in layers in the (100) planes and the shortest inter­molecular contacts are H⋯H (2.30 Å).

## Related literature

The title compound is an inter­mediate in the synthesis of Frovatriptan, a 5-HT_1_-like agonist, see: Borrett *et al.* (1999 ▶). For details of the synthesis, see: Babler & Spina (1984 ▶); Borrett *et al.* (1999 ▶). For a related structure, see: Luger *et al.* (1972 ▶). 
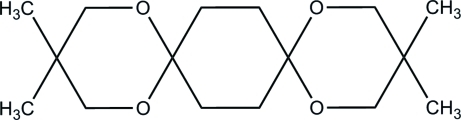


## Experimental

### 

#### Crystal data

C_16_H_28_O_4_
*M* *_r_* = 284.38
Monoclinic, 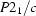

*a* = 12.639 (8) Å
*b* = 5.838 (4) Å
*c* = 11.179 (7) Å
β = 110.611 (8)°
*V* = 772.1 (9) Å^3^
*Z* = 2
Mo *K*α radiation
μ = 0.09 mm^−1^
*T* = 93 K
0.43 × 0.37 × 0.14 mm

#### Data collection

Rigaku SPIDER diffractometer
Absorption correction: none
5721 measured reflections
1752 independent reflections
1243 reflections with *I* > 2σ(*I*)
*R*_int_ = 0.048

#### Refinement

*R*[*F* ^2^ > 2σ(*F* ^2^)] = 0.049
*wR*(*F* ^2^) = 0.095
*S* = 1.00
1752 reflections
93 parameters
H-atom parameters constrained
Δρ_max_ = 0.27 e Å^−3^
Δρ_min_ = −0.19 e Å^−3^

### 

Data collection: *RAPID-AUTO* (Rigaku 2004 ▶); cell refinement: *RAPID-AUTO*; data reduction: *RAPID-AUTO*; program(s) used to solve structure: *SHELXS97* (Sheldrick, 2008 ▶); program(s) used to refine structure: *SHELXL97* (Sheldrick, 2008 ▶); molecular graphics: *SHELXTL* (Sheldrick, 2008 ▶); software used to prepare material for publication: *SHELXTL*.

## Supplementary Material

Crystal structure: contains datablocks I, global. DOI: 10.1107/S1600536809021291/bi2376sup1.cif

Structure factors: contains datablocks I. DOI: 10.1107/S1600536809021291/bi2376Isup2.hkl

Additional supplementary materials:  crystallographic information; 3D view; checkCIF report

## Figures and Tables

**Fig. 1.**
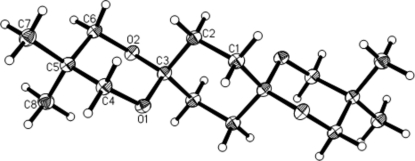
The molecular structure with displacement ellipsoids drawn at the 30% probability level for non-H atoms. Non-labelled atoms are related to labelled atoms by the symmetry operator: 1 - x, 1 - y, 1 - z.

## supplementary crystallographic information

### Comment

The title compound is useful as an intermediate in the synthesis of Frovatriptan, a 5-HT_1_-like agonist (Babler & Spina, 1984; Borrett *et al.*, 1999). The molecular structure is shown in Fig. 1. Three six-membered rings exhibit chair conformations, with C—C bond lengths in the range 1.516 (2)–1.527 (2) Å and C—C—C angles in the range 106.59 (13)–112.55 (13)°; these agree well with the values in other cyclohexane derivatives described in the literature (Luger *et al.*, 1972).

### Experimental

The title compound was obtained by reaction of 1,4-cyclohexanedione (20 mmol), 2,2-dimethyl-1,3-propanediol (40 mmol) and sulfuric acid (0.1 mmol) in hexane (20 ml). The mixture was stirred for 6 h at 333 K. Colourless block-shaped crystals suitable for X-ray diffraction analysis were grown at the bottom of the vessel after 7 days slow evaporation of the solution at room temperature.

### Refinement

H atoms were placed in calculated positions with C—H = 0.99 and 0.98 Å for methylene and methyl H atoms, respectively, and refined as riding with *U*_iso_(H) = 1.2 or 1.5*U*_eq_(C).

### Crystal data

**Table:**

C_16_H_28_O_4_	*F*(000) = 312
*M**_r_* = 284.38	*D*_x_ = 1.223 Mg m^−^^3^
Monoclinic, *P*2_1_/*c*	Melting point: 430 K
Hall symbol: -P 2ybc	Mo *K*α radiation, λ = 0.71073 Å
*a* = 12.639 (8) Å	Cell parameters from 2071 reflections
*b* = 5.838 (4) Å	θ = 3.3–27.5°
*c* = 11.179 (7) Å	µ = 0.09 mm^−^^1^
β = 110.611 (8)°	*T* = 93 K
*V* = 772.1 (9) Å^3^	Block, colourless
*Z* = 2	0.43 × 0.37 × 0.14 mm

### Data collection

**Table:**

Rigaku SPIDER diffractometer	1243 reflections with *I* > 2σ(*I*)
Radiation source: Rotating Anode	*R*_int_ = 0.048
graphite	θ_max_ = 27.5°, θ_min_ = 3.4°
ω scans	*h* = −16→16
5721 measured reflections	*k* = −7→7
1752 independent reflections	*l* = −13→14

### Refinement

**Table:**

Refinement on *F*^2^	Primary atom site location: structure-invariant direct methods
Least-squares matrix: full	Secondary atom site location: difference Fourier map
*R*[*F*^2^ > 2σ(*F*^2^)] = 0.049	Hydrogen site location: inferred from neighbouring sites
*wR*(*F*^2^) = 0.095	H-atom parameters constrained
*S* = 1.00	*w* = 1/[σ^2^(*F*_o_^2^) + (0.0116*P*)^2^ + 0.36*P*] where *P* = (*F*_o_^2^ + 2*F*_c_^2^)/3
1752 reflections	(Δ/σ)_max_ < 0.001
93 parameters	Δρ_max_ = 0.27 e Å^−^^3^
0 restraints	Δρ_min_ = −0.19 e Å^−^^3^

### Special details

**Table:**

Geometry. All e.s.d.'s (except the e.s.d. in the dihedral angle between two l.s. planes) are estimated using the full covariance matrix. The cell e.s.d.'s are taken into account individually in the estimation of e.s.d.'s in distances, angles and torsion angles; correlations between e.s.d.'s in cell parameters are only used when they are defined by crystal symmetry. An approximate (isotropic) treatment of cell e.s.d.'s is used for estimating e.s.d.'s involving l.s. planes.
Refinement. Refinement of *F*^2^ against ALL reflections. The weighted *R*-factor *wR* and goodness of fit *S* are based on *F*^2^, conventional *R*-factors *R* are based on *F*, with *F* set to zero for negative *F*^2^. The threshold expression of *F*^2^ > σ(*F*^2^) is used only for calculating *R*-factors(gt) *etc*. and is not relevant to the choice of reflections for refinement. *R*-factors based on *F*^2^ are statistically about twice as large as those based on *F*, and *R*- factors based on ALL data will be even larger.

### Fractional atomic coordinates and isotropic or equivalent isotropic displacement parameters (Å^2^)

**Table:**

	*x*	*y*	*z*	*U*_iso_*/*U*_eq_	
O1	0.68024 (9)	0.62454 (18)	0.57453 (10)	0.0219 (3)	
O2	0.66546 (9)	0.37232 (18)	0.73088 (10)	0.0221 (3)	
C1	0.43232 (13)	0.7060 (3)	0.48564 (15)	0.0224 (4)	
H1A	0.3649	0.7851	0.4912	0.027*	
H1B	0.4768	0.8194	0.4574	0.027*	
C2	0.50381 (13)	0.6171 (3)	0.61780 (15)	0.0220 (4)	
H2A	0.5299	0.7478	0.6774	0.026*	
H2B	0.4570	0.5166	0.6505	0.026*	
C3	0.60572 (13)	0.4836 (3)	0.61294 (15)	0.0204 (4)	
C4	0.74097 (14)	0.7887 (3)	0.66956 (15)	0.0232 (4)	
H4A	0.6868	0.8977	0.6843	0.028*	
H4B	0.7928	0.8768	0.6380	0.028*	
C5	0.80864 (13)	0.6730 (3)	0.79466 (15)	0.0217 (4)	
C6	0.72591 (14)	0.5256 (3)	0.83191 (15)	0.0222 (4)	
H6A	0.7677	0.4360	0.9093	0.027*	
H6B	0.6715	0.6257	0.8526	0.027*	
C7	0.85930 (15)	0.8559 (3)	0.89653 (16)	0.0284 (4)	
H7A	0.9083	0.9571	0.8691	0.034*	
H7B	0.9038	0.7820	0.9773	0.034*	
H7C	0.7983	0.9459	0.9085	0.034*	
C8	0.90174 (14)	0.5242 (3)	0.77798 (16)	0.0288 (4)	
H8A	0.8680	0.4097	0.7113	0.035*	
H8B	0.9430	0.4466	0.8587	0.035*	
H8C	0.9541	0.6206	0.7532	0.035*	

### Atomic displacement parameters (Å^2^)

**Table:**

	*U*^11^	*U*^22^	*U*^33^	*U*^12^	*U*^13^	*U*^23^
O1	0.0275 (6)	0.0188 (6)	0.0206 (6)	−0.0030 (5)	0.0099 (5)	−0.0001 (5)
O2	0.0292 (6)	0.0154 (6)	0.0197 (6)	−0.0016 (5)	0.0060 (5)	0.0013 (5)
C1	0.0282 (9)	0.0151 (8)	0.0240 (9)	0.0018 (6)	0.0093 (7)	−0.0016 (7)
C2	0.0262 (9)	0.0184 (8)	0.0217 (9)	0.0008 (6)	0.0087 (7)	−0.0019 (7)
C3	0.0254 (9)	0.0167 (8)	0.0188 (8)	−0.0007 (6)	0.0074 (7)	0.0024 (7)
C4	0.0289 (9)	0.0160 (8)	0.0240 (9)	−0.0042 (7)	0.0083 (7)	0.0000 (7)
C5	0.0258 (9)	0.0182 (8)	0.0209 (9)	−0.0010 (7)	0.0083 (7)	0.0002 (7)
C6	0.0277 (9)	0.0207 (8)	0.0175 (9)	0.0002 (7)	0.0073 (7)	0.0003 (7)
C7	0.0331 (10)	0.0254 (9)	0.0253 (10)	−0.0041 (8)	0.0084 (8)	−0.0010 (8)
C8	0.0281 (9)	0.0291 (10)	0.0293 (10)	0.0016 (7)	0.0105 (8)	0.0008 (8)

### Geometric parameters (Å, °)

**Table:**

O1—C3	1.4258 (19)	C4—H4A	0.990
O1—C4	1.4362 (19)	C4—H4B	0.990
O2—C3	1.4248 (19)	C5—C6	1.521 (2)
O2—C6	1.4328 (19)	C5—C8	1.526 (2)
C1—C3^i^	1.516 (2)	C5—C7	1.527 (2)
C1—C2	1.526 (2)	C6—H6A	0.990
C1—H1A	0.990	C6—H6B	0.990
C1—H1B	0.990	C7—H7A	0.980
C2—C3	1.523 (2)	C7—H7B	0.980
C2—H2A	0.990	C7—H7C	0.980
C2—H2B	0.990	C8—H8A	0.980
C3—C1^i^	1.516 (2)	C8—H8B	0.980
C4—C5	1.517 (2)	C8—H8C	0.980
			
C3—O1—C4	113.58 (12)	H4A—C4—H4B	108.0
C3—O2—C6	113.98 (12)	C4—C5—C6	106.59 (13)
C3^i^—C1—C2	112.55 (13)	C4—C5—C8	110.46 (14)
C3^i^—C1—H1A	109.1	C6—C5—C8	110.18 (14)
C2—C1—H1A	109.1	C4—C5—C7	109.17 (14)
C3^i^—C1—H1B	109.1	C6—C5—C7	109.84 (14)
C2—C1—H1B	109.1	C8—C5—C7	110.50 (14)
H1A—C1—H1B	107.8	O2—C6—C5	111.39 (13)
C3—C2—C1	111.07 (13)	O2—C6—H6A	109.3
C3—C2—H2A	109.4	C5—C6—H6A	109.3
C1—C2—H2A	109.4	O2—C6—H6B	109.3
C3—C2—H2B	109.4	C5—C6—H6B	109.3
C1—C2—H2B	109.4	H6A—C6—H6B	108.0
H2A—C2—H2B	108.0	C5—C7—H7A	109.5
O2—C3—O1	110.54 (12)	C5—C7—H7B	109.5
O2—C3—C1^i^	105.59 (13)	H7A—C7—H7B	109.5
O1—C3—C1^i^	106.05 (13)	C5—C7—H7C	109.5
O2—C3—C2	112.32 (13)	H7A—C7—H7C	109.5
O1—C3—C2	111.86 (13)	H7B—C7—H7C	109.5
C1^i^—C3—C2	110.12 (13)	C5—C8—H8A	109.5
O1—C4—C5	111.45 (13)	C5—C8—H8B	109.5
O1—C4—H4A	109.3	H8A—C8—H8B	109.5
C5—C4—H4A	109.3	C5—C8—H8C	109.5
O1—C4—H4B	109.3	H8A—C8—H8C	109.5
C5—C4—H4B	109.3	H8B—C8—H8C	109.5
			
C3^i^—C1—C2—C3	56.01 (19)	C1—C2—C3—C1^i^	−54.63 (18)
C6—O2—C3—O1	54.99 (16)	C3—O1—C4—C5	57.12 (17)
C6—O2—C3—C1^i^	169.25 (12)	O1—C4—C5—C6	−54.38 (17)
C6—O2—C3—C2	−70.72 (16)	O1—C4—C5—C8	65.32 (17)
C4—O1—C3—O2	−55.06 (16)	O1—C4—C5—C7	−172.97 (13)
C4—O1—C3—C1^i^	−169.04 (12)	C3—O2—C6—C5	−56.61 (17)
C4—O1—C3—C2	70.90 (16)	C4—C5—C6—O2	54.05 (17)
C1—C2—C3—O2	−172.00 (12)	C8—C5—C6—O2	−65.83 (17)
C1—C2—C3—O1	63.02 (16)	C7—C5—C6—O2	172.21 (13)

Symmetry codes: (i) −*x*+1, −*y*+1, −*z*+1.
